# Effect of Arabinoxylan and Xylo-Oligosaccharide on Growth Performance and Intestinal Barrier Function in Weaned Piglets

**DOI:** 10.3390/ani13060964

**Published:** 2023-03-07

**Authors:** Feize Sun, Huahui Li, Zhiqiang Sun, Ling Liu, Xiujun Zhang, Jinbiao Zhao

**Affiliations:** 1 School of Public Health, North China University of Science and Technology, Tangshan 063210, China; 2 State Key Laboratory of Animal Nutrition, College of Animal Science and Technology, China Agricultural University, Beijing 100193, China

**Keywords:** xylo-oligosaccharides, arabinoxylans, weaned piglets, gut microbiota, intestinal barrier

## Abstract

**Simple Summary:**

The study was conducted to explore effects of xylose with different polymerizations on growth performance, intestinal barrier function, and gut microbial composition in weaned piglets. Results showed that dietary supplementation of 1% xylo-oligosaccharide (XOS) or 1% arabinoxylan (AX) reduced diarrhea incidence of piglets compared with the control group (CON) but increased intestinal villus height, antioxidase activity, and sIgA contents (*p* < 0.05). With in vitro fermentation assay, XOS showed a sharper reduction of the pH curve than AX (*p* < 0.05). XOS increased the abundance of *Lactobacillus* and *Bifidobacterium* in the ileal digesta (*p* < 0.05), while AX increased the abundance of *Bifidobacterium* in the colonic digesta (*p* < 0.05). In conclusion, both XOS and AX improved the intestinal barrier function and reduced the diarrhea incidence of weaned piglets, and the XOS showed a faster microbial fermentation than the AX due to a lower polymerization and molecular mass.

**Abstract:**

The purpose of this study was to explore the effects of xylose with different polymerizations on growth performance, intestinal barrier function, and gut microbial composition in weaned piglets. A total of 144 weaned piglets were assigned to 3 dietary treatments in a completely randomized design according to their body weight and sex. Dietary treatments included a corn-soybean meal basal diet (CON) and 2 additional diets formulated with 1% arabinoxylan (AX) and 1% xylo-oligosaccharide (XOS), respectively. Results showed that dietary supplementation of XOS or AX reduced diarrhea incidence of weaned piglets compared with the CON group (*p* < 0.05). XOS or AX increased the ileal villus height and intestinal activity of antioxidases in weaned piglets compared with the CON group (*p* < 0.05). XOS or AX reduced the ileal and colonic IL-6 content and increased the colonic sIgA and IL-10 concentrations in weaned piglets compared with the CON group (*p* < 0.05). XOS or AX increased the total organic acids concentrations in the ileum and in vitro fermentation (*p* < 0.05). XOS increased the abundance of *Lactobacillus* and *Bifidobacterium* in the ileal digesta (*p* < 0.05), while AX increased the population of *Lactobacillus* in the ileal digesta and the abundance of *Bifidobacterium* in the colonic digesta of weaned piglets (*p* < 0.05). In conclusion, both XOS and AX reduce diarrhea incidence and improve antioxidant capacity, immune function, and populations of beneficial bacteria, while microbial fermentation of XOS with a lower polymerization and molecular mass can produce more organic acids and an increased abundance of *Lactobacillus* and *Bifidobacterium* in the upper gut of weaned pigs compared with AX.

## 1. Introduction

Early weaning of young animals would damage the intestinal function of the host due to the severe oxidation stress derived from changes of diet and environment, resulting in diarrhea incidence and loss of appetite [1,2]. Early weaning of piglets is a necessary strategy for intensive production in modern pig farming, and it has been reported that early weaning always results in a series of problems such as increased diarrhea, poor feed utilization, and low survival rate since weaning stress can disrupt the intestinal redox balance and immune function of piglets [3,4]. Numerous studies have shown that dietary fiber has an important role in improving the intestinal barrier function of the host [5] and maintaining intestinal environmental homeostasis by regulating gut microbial composition [6]. Dietary fiber as forms of polysaccharides can be degraded by gut microbiota into oligosaccharides, including fructo-oligosaccharides, isomalto-oligosaccharides, galacto-oligosaccharides, and xylo-oligosaccharides (XOS) [7]. Polysaccharides primarily consist of resistant starch, cellulose, and hemicellulose. Arabinoxylan (AX) is one of the most abundant hemicellulose in the cell wall of cereal plants, which can be further degraded into XOS by gut microbiota of the host to produce short chain fatty acids (SCFA) [8]. Generally, XOS are connected with the xylose with 2–7 glycosidic bonds and the backbone chain of AX is connected with the xylose with more than 10 glycosidic bonds [9,10]. Recent studies have shown that AX and XOS can act as prebiotic substances with positive effects on maintaining the intestinal health of the host. In addition, AX and XOS in wheat bran have a beneficial effect on the balance of intestinal microorganisms in the in vitro fermentation assay of humans [11]. Similarly, the addition of XOS to the diet of weaned piglets can improve the intestinal barrier function, reduce inflammatory responses, and regulate the microbial community of piglets [4]. The XOS also showed similar benefits on increasing growth performance and improving gastrointestinal microbiota composition in broilers [12].

However, the differences of XOS and AX with varying fermentation rates and molecular weights in regulating growth performance, intestinal barrier function, and microbial composition have been unclear. Therefore, this study aimed to explore the effects of AX and XOS supplemented in the diet on growth performance, diarrhea incidence, nutrient digestibility, intestinal morphology, antioxidant capacity, immune function, and gut microbiota in weaned piglets.

## 2. Materials and Methods

### 2.1. Sources of XOS and AX

The XOS used in the study were extracted from corn stover with a purity of 70% and brought from Shandong Bailongchuangyuan Biotechnology Co., Ltd. (Dezhou, China), and the primary components of XOS are xylose connecting with 2–4 glycosidic bonds. In addition, the AX was extracted from corn stalks followed by a protocol as described before [13]. Briefly, fat was removed by suspending corn bran in hexane (1:10, *w*/*v*) and stirring for 30–45 min. The slurry was filtered, and the process was repeated once. The final residual was dried in a hot air oven at 45 °C. Then, starch and protein were removed using thermostable α-amylase and proteinase, respectively, to obtain de-starched bran. The bran was suspended in 1 M sodium hydroxide solution (1:10, *w*/*v*). After stirring for 24 h at room temperature, the suspension was centrifuged at 14,000 rpm for 10 min (5424R, Eppendorf Co., Ltd., Hamburg, Germany). The supernatant pH was adjusted to 4–5 and 4 volumes of ethanol was added. The precipitate was dried in a hot air oven at 45 °C, re-dissolved, and freeze-dried to obtain AX with a purity of 85%.

### 2.2. Animals, Diets, and Experimental Design

The feeding trial was conducted at the animal testing base in the National Feed Engineering Technology Research Center of China Agricultural University (Fengning, Hebei, China). A total of 144 Duroc × (Landrace × Large White) weaned piglets with an initial average weight of 7.24 ± 0.19 kg and age of 28 ± 3 d were assigned to 3 dietary treatments according to their body weight and sex in a completely randomized design. Dietary treatments included a corn-soybean meal basal diet (CON) and 2 additional diets formulated with 1% AX and 1% XOS based on the same control diet, respectively. Each treatment included 8 replicates (pens) with 6 pigs (3 barrows and 3 gilts) in each replicate. Premixes of trace minerals and vitamins were supplemented in the diets to meet the nutrient requirements of weaned pigs (NRC, 2012). The digestible energy and standardized ileal digestibility of amino acids were equal among all dietary treatments. Ingredients composition and nutritive level in the diets are shown in Supplementary Table S1. No medicine and antibiotics were used in the whole feeding trial.

This feeding trial lasted 28 d, and the pigs were provided ad libitum access to water and feed. All pigs were individually housed in a pen (1.5 m × 1.2 m × 0.8 m) equipped with a nipple drinker and a feeder. The humidity and temperature were controlled at 50%–60% and 25 °C–28 °C, respectively. The pigs were housed in pens with half cement floor and half woven mesh floor.

### 2.3. Sample Collection

Fecal samples (300 g) from each pen (n = 6, 6 pens per treatment) were collected at 26–27 d in the feeding trial and mixed before drying. Experimental diets and fecal samples were oven dried at 65 °C for 72 h. The dried fecal samples were rewarmed for 24 h to a constant weight, crushed, and passed through a 60 mesh sieve and bagged for use. On the 28th day of the experiment, one piglet near the average body weight was selected from each pen for euthanasia, and samples of tissues and digesta from the ileum and colon of the piglets were collected. Appropriate amounts of intestinal tissues and digesta were immediately placed in liquid nitrogen and subsequently stored at −80 °C. The jejunum and colonic tissues were taken simultaneously, and the intestinal digestive contents were removed and then preserved in 4% paraformaldehyde fixative.

### 2.4. Chemical Analysis and Calculation

#### 2.4.1. Growth Performance

The body weight of the piglets was recorded one by one on day 0, 14, and 28 to calculate the average daily gain (ADG), and the feed consumption was recorded for each replicate (pen) on day 14 and 28 to calculate the average daily feed intake (ADFI). A ratio of average daily feed intake to average daily weight gain was calculated and expressed as the feed conversion ratio (FCR). The diarrhea situation of each piglet was observed daily during the feeding trial, and the diarrhea incidence was calculated by referring to a previous study [14]; the criteria for fecal score were as follows: grade 1, hard, dry, and friable feces; grade 2, normal feces; grade 3, pasty feces, mild diarrhea; grade 4, unformed feces, moderate diarrhea; grade 5, watery feces, severe diarrhea. (Supplementary Table S2). The pig was considered positive for clinical symptoms of diarrhea when the score was over 3.

Diarrhea incidence (%) = (number of days with diarrhea symptoms in piglets/total number of days in the test) × 100.

#### 2.4.2. Nutrient Digestibility

Gross energy (GE) in the samples of diets and feces was determined according to the ISO 9831:1998 method using an oxygen bomb calorimeter (Parr 6300 Calorimeter, Moline, IL, USA), and neutral detergent fiber (NDF) and acid detergent fiber (ADF) were determined using a fiber analyzer (ANKOM^200^) according to the method of van Soest et al. [15]. Acid insoluble ash (AIA) in the diets and feces was measured as described by De Coca-Sinova et al. [16]. Apparent total tract digestibility of the nutrient was determined using acid insoluble ash as an indicator. Dry matter (DM; method 934.01) and crude protein (CP; method 990.03) in the samples of diets and feces was analyzed according to the procedures of the Association of Official Analytical Chemists [17], and samples of diets were analyzed for SDF (method 991.43) and IDF (method 991.43).

#### 2.4.3. Intestinal Morphology

The ileal and colonic tissues in fixative were taken, paraffin sections were made, and ileal morphology was analyzed using hematoxylin-eosin (H&E) staining. The intestinal morphological structures were observed under a 10× field of view using a light microscope (Japan, OLYMPUS), and the villi height and crypt depth were randomly measured under 10 different fields of view, and the mean values were calculated.

#### 2.4.4. Intestinal Antioxidant and Immune Function

About 100 mg of ileal and colonic samples were weighed and placed in 1 mL PBS to prepare homogenate, centrifuged at 4000 rpm for 10 min at 4 °C (5424R, Eppendorf Co., Ltd., Hamburg, Germany), and the supernatant was collected. Superoxide dismutase (SOD), malondialdehyde (MDA), glutathione peroxidase (GSH-Px), and total antioxidant capacity (T-AOC) activities in the intestinal epithelium were measured using a semi-automatic biochemical analyzer (A6, Songshang Technology Co., Ltd., Beijing, China). Concentrations of secretory immunoglobulin A (sIgA), interleukin 2 (IL-2), interleukin 6 (IL-6), interleukin 10 (IL-10), interleukin 12 (IL-12), interleukin 1β (IL-1β), and tumor necrosis factor α (TNF-α) in the ileal and colonic mucosa were determined using an enzyme-labeled instrument. (Huawei Delang DR-200BS, Huawei Delang Instrument Co., Ltd., Wuxi, China).

### 2.5. In Vitro Fermentation Assay

The intestinal digesta were collected and frozen and sent to the in vitro fermentation laboratory for processing. Under aseptic conditions, 100 g of digesta was weighed into 500 mL of saline for each group, stirred well, and filtered using four layers of medical sterile gauze. Using in vitro fermentation assay, the buffer solution was formulated with 8.32 g/L NaHCO_3_, 0.95 g/L NH_4_HCO_3_, 1.36 g/L Na_2_HPO_4_, 1.47 g/L KH_2_PO_4_, 0.14 g/L MgSO_4_·7H_2_O, 0.30 g/L Na_2_S_9_H_2_O, 76.09 mg/L NaOH, 15.69 mg/L CaCl_2_·2H_2_O, 11.89 mg/L MnCl_2_·4 H_2_O, 1.19 mg/L CoCl_2_·6H_2_O, 9.51 mg/L FeCl_3_·6H_2_O, and 1.19 mg/L resazurin. The medium and fermentation broth were mixed according to the ratio of 11:1, and 0.5 g of XOS and AX were weighed as two fermentation substrates, respectively, and incubated in an incubator at 37 ± 0.5 °C for 48 h. The supernatant of fermentation broth was collected via centrifugation at 12,000 rpm for 10 min (5424R, Eppendorf Co., Ltd., Hamburg, Germany) for the determination of SCFA concentrations.

### 2.6. Determination of SCFA Concentration

About 1 g of digesta (1 mL of in vitro fermentation broth) was weighed in 8 mL of ultrapure water, sonicated for 30 min, shaken every 10 min, and centrifuged at 5000 rpm for 10 min (5424R, Eppendorf Co., Ltd., Hamburg, Germany). After 50-fold dilution, 1.5 mL of sample was taken and filtered using a 0.22 μm membrane (Millipore, Bedford, OH, USA) and injected into an ionization. The chromatographic column was an ICS-3000 (250 mm × 4 mm).

### 2.7. Intestinal Microbial Community

About 10 g of samples were blended under CO_2_ in 90 mL of anaerobic dilution solution. Quadruplicate plates were then inoculated with 0.1 mL samples and incubated at 37 °C anaerobically. Three dilutions were plated for each medium. Bacteria were enumerated on MRS agar (Oxoid; *Lactobacillus*) and reinforced clostridial agar plus supplements (Munoa and Pares; *Bifidobacterium*). Single colonies were removed from selective media plates and grown in peptone yeast glucose (PYG) broth [18]. Subsequently, the bacteria were characterized to genus level on the basis of colonial appearance, gram reaction, spore production, cell morphology, and fermentation end-product formation [19].

### 2.8. Statistical Analysis

All data were analyzed using ANOVA with the GLM model of SAS 9.4 (SAS Institute, Cary, NC, USA). Normality was verified and outliers were identified using the UNIVARIATE procedure of SAS 9.4. An observation was considered an outlier if the value was more than 3 standard deviations away from the grand mean, and no outliers were observed in the study. A replicate (pen) was analyzed as an experimental unit. Dietary treatment was considered as the fixed effect and the animal was the random effects. Mean values were calculated using the LSMEANS procedure. Statistical differences were separated via Tukey’s multiple range test. The significance level was set at *p* < 0.05, whereas 0.05 ≤ *p* < 0.10 was considered as a tendency.

## 3. Results

### 3.1. Effect of AX and XOS on Growth Performance and Diarrhea Incidence of Weaned Piglets

**Figure 1:**
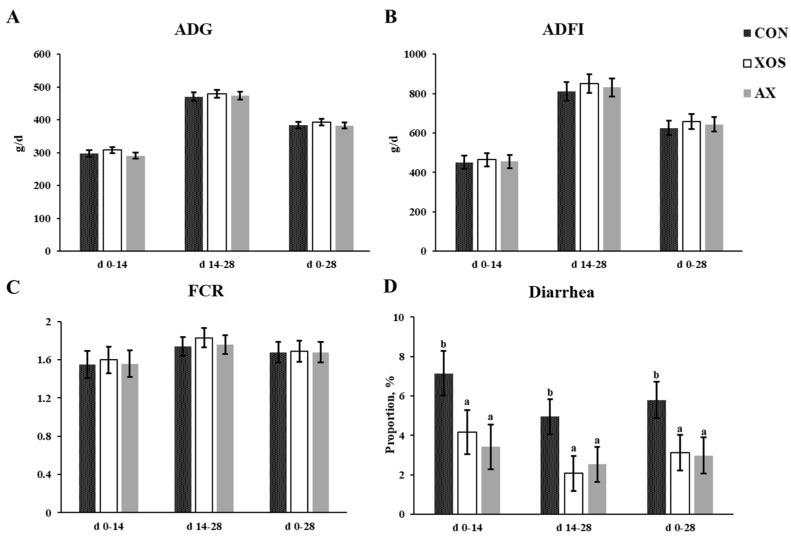
Effects of xylo-oligosaccharides and arabinoxylans on growth performance of weaned pigs. Note: (**A**) ADG, average daily gain; (**B**) ADFI, average daily feed intake; (**C**) FCR, feed conversion ratio; (**D**) diarrhea. Note: a, b, significant differences (*p* < 0.05) between different letters in the same row of shoulder labels (n = 6); CON, a control diet; XOS, a diet containing 1% xylo-oligosaccharide; AX, a diet containing 1% arabinoxylans; SEM, standard error of the mean.

As shown in Figure 1, the addition of 1% XOS or 1% AX to the diet had no significant effects on body weight, ADG, ADFI, and FCR of weaned piglets throughout the whole trial period (*p* > 0.1). However, the addition of XOS and AX to the diet significantly reduced the diarrhea incidence of weaned piglets compared with the control group (*p <* 0.05). The difference in diarrhea incidence of weaned piglets was not significantly different between the XOS and the AX groups.

### 3.2. Effect of AX and XOS on Nutrients Digestibility in Weaned Piglets

**Figure 2:**
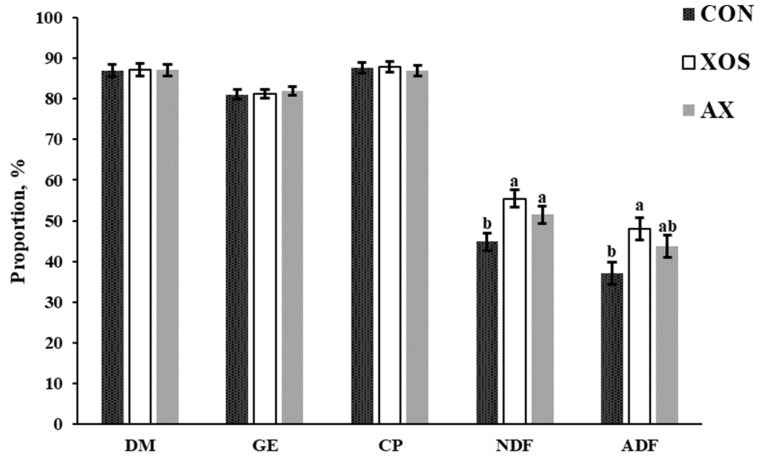
Effects of xylo-oligosaccharides and arabinoxylans on apparent total tract digestibility of dietary nutrients in weaned pigs. Note: a,b significant differences (*p* < 0.05) between different letters in the same row of shoulder labels (n = 6); CON, a control diet; XOS, a diet containing 1% xylo-oligosaccharide; AX, a diet containing 1% arabinoxylans; SEM, standard error of the mean. DM, dry matter; GE, gross energy; CP, crude protein; NDF, neutral detergent fiber; ADF, acid detergent fiber.

As shown in Figure 2, the addition of XOS to the diet significantly increased the digestibility of NDF and ADF in weaned piglets compared with the CON group (*p* < 0.05). The digestibility of NDF in the AX group also increased significantly (*p <* 0.05), while the difference in the digestibility of ADF was not observed compared with the CON group (*p* > 0.05). In addition, the addition of AX or XOS to the diet had no significant effects on the ATTD of DM, GE, and CP.

### 3.3. Effect of AX and XOS on Intestinal Morphology in Weaned Piglets

**Figure 3:**
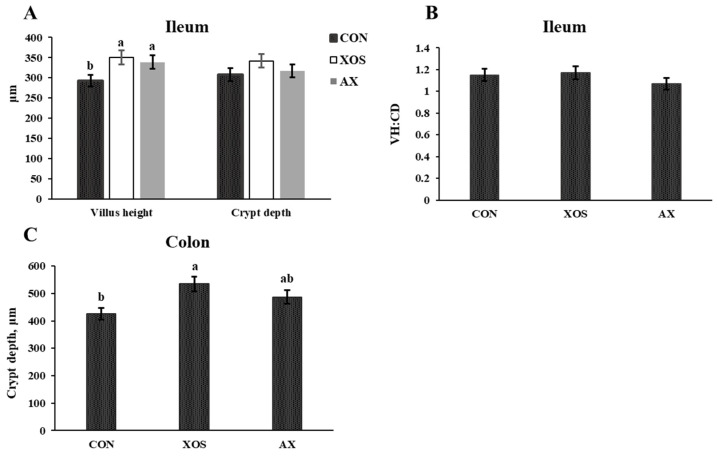
Effects of xylo-oligosaccharides and arabinoxylans on intestinal morphology in the ileum and colon of weaned pigs. Note: (**A**) villus height and crypt depth of the ileum; (**B**) VH: CD of the jejunum; (**C**) crypt depth of the colon. a, b Significant differences (*p* < 0.05) between different letters in the same row of shoulder labels (n = 6); CON, a control diet; XOS, a diet containing 1% xylo-oligosaccharide; AX, a diet containing 1% arabinoxylans; SEM, standard error of the mean.

As shown in Figure 3, the ileal villus height in the XOS and AX groups was significantly greater than those in the CON group, and the colonic crypt depth was significantly higher in the XOS group compared with the AX and CON groups (*p* < 0.05). However, the addition of XOS or AX to the diet had no significant effects on the crypt depth and villous crypt ratio in the ileum of weaned piglets (*p* > 0.05).

### 3.4. Effect of AX and XOS on Intestinal Antioxidant Capacity in Weaned Piglets

**Figure 4:**
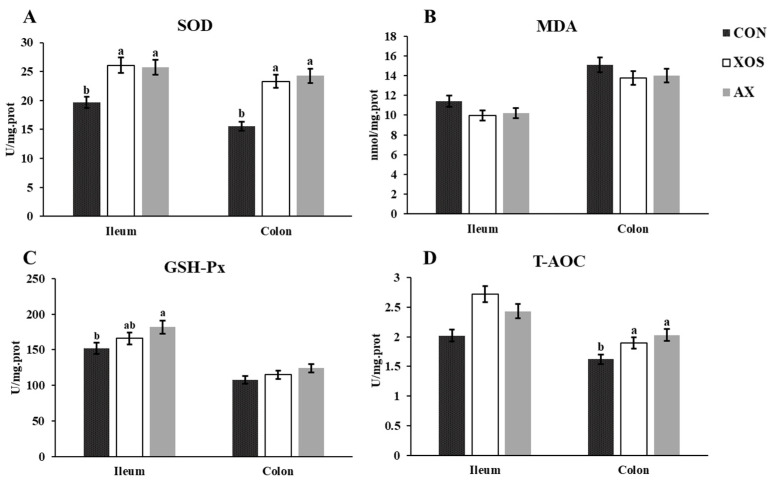
Effects of xylo-oligosaccharides and arabinoxylans on intestinal antioxidants of weanling pigs. Note: (**A**) superoxide dismutase (SOD); (**B**) malondialdehyde (MDA); (**C**) glutathione peroxidase (GSH-Px); (**D**) total antioxidant capacity (T-AOC). a, b Significant differences (*p* < 0.05) between different letters in the same row with shoulder labels (n = 6); CON, a control diet; XOS, a diet containing 1% xylo-oligosaccharide; AX, a diet containing 1% arabinoxylans; SEM, standard error of the mean.

As shown in Figure 4, the addition of XOS or AX to the diet significantly increased the activity of SOD and GSH-P_X_ in the ileum of weaned piglets compared with the CON group (*p* < 0.05). The colonic SOD and T-AOC activities in the XOS and AX groups were significantly greater than those in the CON group (*p* < 0.05).

### 3.5. Effect of AX and XOS on Intestinal Immune Function in Weaned Piglets

**Figure 5:**
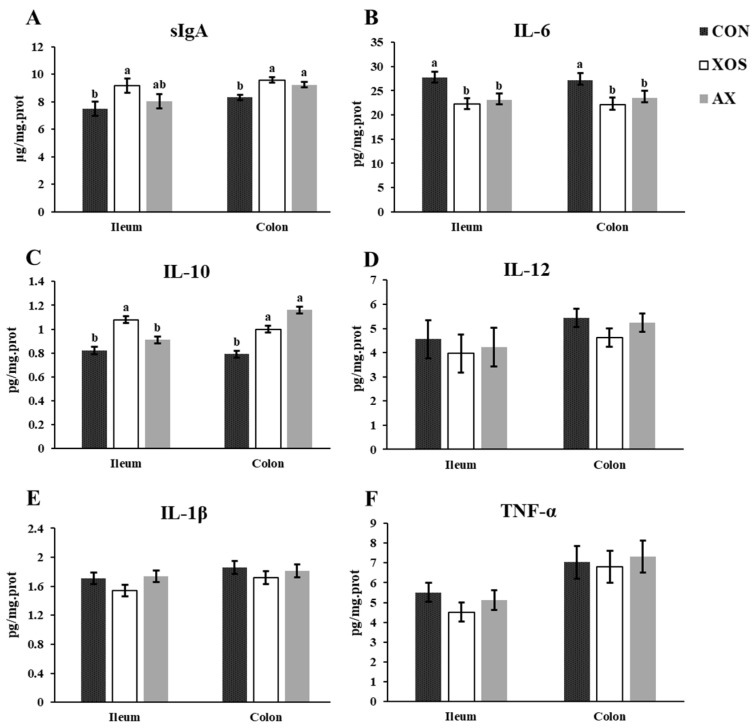
Effects of xylo-oligosaccharides and arabinoxylans on intestinal immune function of weaned piglets. Note: (**A**) secretory immunoglobulin A (sIgA); (**B**) interleukin 6 (IL-6); (**C**) interleukin 10 (IL-10); (**D**)interleukin 12 (IL-12); (**E**) interleukin 1β (IL-1β); (**F**) tumor necrosis factor α (TNF-α). a, b Significant differences (*p* < 0.05) between different letters in the same row of shoulder labels (n = 6); CON, a control diet; XOS, a diet containing 1% xylo-oligosaccharide; AX, a diet containing 1% arabinoxylans; SEM, standard error of the mean.

As shown in Figure 5, the addition of XOS or AX to the diet significantly reduced the ileal and colonic IL-6 levels and significantly increased the colonic sIgA and IL-10 concentrations in weaned piglets compared with the CON group (*p* < 0.05). The ileal sIgA and IL-10 in the XOS group were significantly greater than those in the CON group (*p* < 0.05). However, there were no differences in the concentrations of sIgA and inflammatory cytokines, except for the ileal IL-10 content, between the XOS group and the AX group (*p* > 0.05).

### 3.6. Effect of AX and XOS on SCFA Concentrations in the Intestine of Weaned Piglets

**Figure 6:**
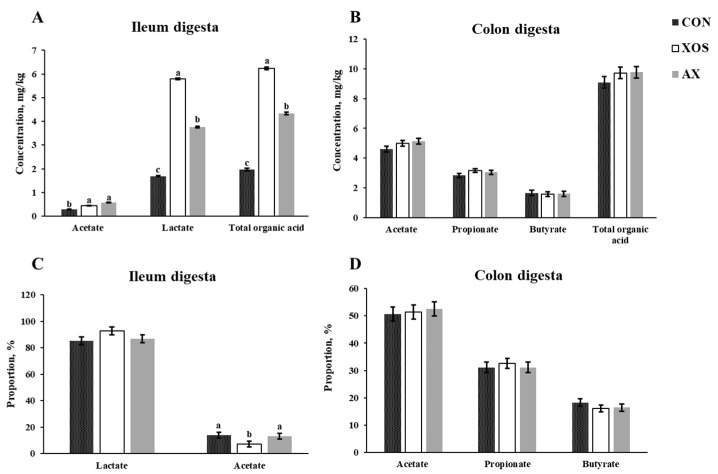
Effects of xylo-oligosaccharides and arabinoxylans on content of organic acids in the intestine of weaned pigs. Note: (**A**) ileum digesta; (**B**) colon digesta; (**C**) ileum digesta; (**D**) colon digesta. a–c Significant differences (*p* < 0.05) between different letters in the same row of shoulder labels (n = 6); CON, a control diet; XOS, a diet containing 1% xylo-oligosaccharide; AX, a diet containing 1% arabinoxylans; SEM, standard error of the mean.

As shown in Figure 6, the addition of XOS or AX to the diet significantly increased the contents of lactic acid, acetic acid, and total organic acids in the ileum of weaned piglets and significantly increased the proportion of acetic acid to total organic acids compared with the CON group (*p* < 0.05). Furthermore, the diet supplemented with 1% XOS significantly increased the lactic acid concentration in the ileum compared with the AX (*p* < 0.05). However, there were no differences in concentrations of lactic acid, acetic acid, propionic acid, butyric acid, and total organic acids in the colonic digesta of weaned piglets among all dietary groups (*p* > 0.05).

### 3.7. Effect of In Vitro Fermentation of AX and XOS on Organic Acid Concentration in the Intestinal Digesta of Weaned Piglets

**Figure 7:**
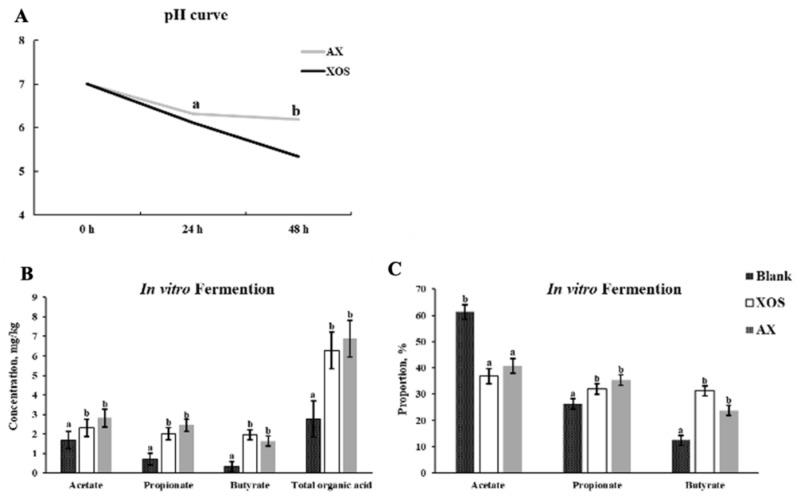
Effects of in vitro fermentation of xylo-oligosaccharides and arabinoxylans on pH and SCFA concentrations. Note: (**A**) pH; (**B**) in vitro fermentation of SCFA concentration; (**C**) in vitro fermentation of SCFA proportion. a, b Significant differences (*p* < 0.05) between different letters in the same row of shoulder labels (n = 6); XOS, xylo-oligosaccharide; AX, arabinoxylans; SEM, standard error of the mean.

As shown in Figure 7, compared with the blank group, AX or XOS significantly increased the contents of acetic acid, propionic acid, butyric acid, and total organic acids in vitro fermentation using the colonic digesta of weaned piglets (*p* < 0.05). However, the proportion of acetic acid to total organic acid was greater in the control group than that in the other groups, and proportions of propionic acid and butyric acid to total organic acid were greater in the AX and XOS groups than those in the control group (*p* < 0.05). No differences in the concentrations and proportions of organic acids were observed between the XOS and AX groups.

### 3.8. Effect of AX and XOS on Intestinal Microflora of Weaned Piglets

**Figure 8:**
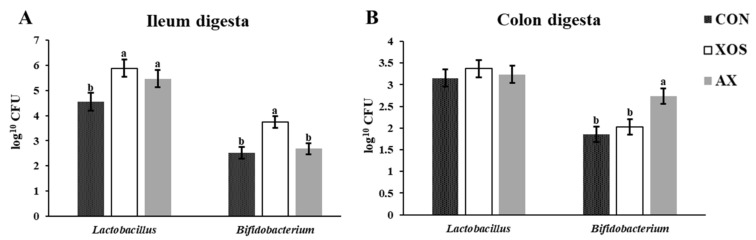
Effects of xylo-oligosaccharides and arabinoxylans on gut microbiota community in weaned pigs. Note: (**A**) ileum digesta; (**B**) colon digesta. a, b Significant differences (*p* < 0.05) between different letters in the same row of shoulder labels (n = 6); CON, a control diet; XOS, a diet containing 1% xylo-oligosaccharide; AX, a diet containing 1% arabinoxylans; SEM, standard error of the mean.

As shown in Figure 8, the addition of XOS to the diet significantly increased the abundance of *Lactobacillus* and *Bifidobacterium* in the ileal digesta of weaned piglets (*p* < 0.05), while the addition of AX to the diet significantly increased the abundance of *Lactobacillus* in the ileal digesta and the abundance of *Bifidobacterium* in the colonic digesta of weaned piglets (*p* < 0.05).

## 4. Discussion

This study showed that the addition of 1% AX or 1% XOS to the diet did not affect the growth performance of weaned piglets compared with the CON group, which is consistent with a previous study [5]. A previous study also found that the addition of XOS-rich crude hydrolysis products to the diet significantly improved ADG and F:G in weaned piglets [6]. However, the results in the previous study showed that the addition of 500 mg/kg XOS to the diet significantly improved the BW, ADG, and F:G ratio in weaned piglets [2]. The above previous studies suggest that whether AX and XOS exert a beneficial effect on growth performance of weaned piglets may be related to the inclusion levels and extraction processing. In addition, significant differences in growth performance of pigs between AX and XOS groups was not observed, which indicated that varying fermentation characteristics of XOS and AX did not affect weaned piglets’ performance. Weaning-induced diarrhea in piglets is a common and highly prevalent gastro-intestinal disorder that negatively affects the immune system and triggers intestinal dysfunction in piglets [20,21]. In our study, both AX and XOS significantly reduced the incidence of diarrhea in weaned piglets. Similarly, Su found that the addition of 0.25 g/kg of XOS to weaned piglet diets significantly reduced the incidence of piglet diarrhea [22], and a previous study showed that use of AX alone or a combination of AX, arabinofuranosidase, and feruloyl esterase can significantly reduce diarrhea incidence in piglets [23], indicating that both XOS and AX have prebiotic effects on reducing diarrhea incidence of weaning piglets.

In the present study, we found that the addition of AX or XOS to the diet had no significant effect on DM, GE, and CP of ATTD, which is inconsistent with previous studies [24]. Similarly, AX or XOS can increase the ATTD of NDF and ADF in piglets. A previous study showed that the ATTD of the NDF and ADF in coarse corn bran for growing pigs was higher than those when the pigs were fed a fine corn bran diet [25]. The improved digestibility of fiber components when the pigs were fed corn bran, AX, and XOS could be associated with the increased microbial diversity in the intestine. In addition, the AX or XOS has a higher ferment ability by the gut microbiota in pigs, which leads to higher levels of produced SCFA and a low pH value to promote development of the intestinal morphology and the immune system, which improved fiber components digestibility [26,27].

In the present study, the addition of XOS or AX to the diet was found to increase the ileal villus height of piglets, which is consistent with a previous study whereby dietary supplementation of 100 mg/kg XOS increased the ileal villus height of piglets [21]. In addition, adding 0.5% XOS in the diet also increased the ileal villus height of broilers in a previous study [28]; however, a previous study reported that dietary supplementation of AX did not affect the villus height in the intestine of piglets [29]. The mentioned results above indicate that XOS has stable and better effects on improving the villus height of the pigs. In our study, the addition of 1% XOS to the diet increased the colonic crypt depth in the piglets, which is consistent with a previous report that found that the ileal crypt depth was significantly higher when the pigs were fed a diet containing 500 mg/kg XOS compared with the CON group [2]. Some studies have shown that XOS can increase the ratio of villus height to crypt depth in the intestine of weaned piglets [22,24]; however, no difference in the ratio of villus height to crypt depth was found after XOS and AX supplementation in the present study.

In the present study, both XOS and AX supplementied into the diet increased activity of SOD and GSH-Px in the ileum of weaned piglets compared with the CON group. Similarly, the colonic SOD and T-AOC activities in the XOS and AX groups were significantly greater than those in the CON group. Our findings were consistent with the previous study in which the serum GSH-Px concentration increased linearly with increasing dietary supplementation levels of XOS from 0% to 3% in weaning pigs [30]. Dietary supplementation of 400 mg/kg XOS decreased the serum MDA level and increased the activity of GSH-Px [31]. Chen et al. also reported that weaning piglets fed a diet containing 500 mg/kg XOS had higher T-SOD and CAT levels on day 28, whereas the MDA level was lower than the control group [2]. A recent report found that maternal supplementation of 500 mg/kg XOS in the diet resulted in a lower concentration of MDA in suckling pigs, although no differences in activity of SOD, T-AOC, and CAT were observed [32]. The above results indicate that dietary supplementation of XOS is beneficial to an improvement of antioxidase activities, but the role of AX in regulating antioxidant capacity should be explored using many further studies.

Numerous studies indicated that XOS can alleviate an inflammatory status in animals. In the present study, both XOS and AX added in the diet reduced ileal and colonic concentrations of IL-6 and increased colonic sIgA and IL-10 concentrations in weaned piglets compared with the CON group. As reported, sow supplementation of 500 mg/kg XOS decreased serum IL-1β, IL-6, IL-2, and TNF-α levels in suckling piglets [2]. In another previous study, dietary supplementation of 1.5 and 3% XOS decreased the serum concentration of IL-1β compared with the control group [30]. The piglets in the XOS-treated group had a lower serum concentration of IL-6 and increased IL-10 content when the piglets were fed a diet containing 1.5% XOS [30]. Yin et al. found that XOS at 100 mg/kg of the feed only decreased the serum concentration of IFN-γ in weaning piglets, but had no impacts on IL-1β, IL-6, and IL-10 [4]. A previous study showed that a diet supplemented with 10% XOS down-regulated the IFN-γ and IL-1β in the mice [33] and XOS suppressed the pro-inflammatory cytokines, IL-1β, IL-6, and TNF-α secretion from RAW264.7 macrophages stimulated with lipopolysaccharide in the pretreatment model [34]. The possible mechanism responsible for the beneficial effects is that XOS can enhance the immune function and decrease the inflammatory status in weaning piglets.

It has been widely demonstrated that XOS can modulate gut microbiota composition by selectively stimulating growth of beneficial bacteria. Many previous studies have reported that dietary supplementation of XOS decreased the diversity of the gut microbiota community [2,4]. The PCoA plots based on Bray–Curtis distances showed a clear separation between the XOS and control groups [30]. It has been reported that XOS can promote counts of *Lactobacillus* in weaning piglets [22,24], while the low level of XOS failed to stimulate the abundance of *Bifidobacterium* [22,30,35]. In our study, the addition of XOS to the diet increased the abundance of *Lactobacillus* and *Bifidobacterium* in the ileal digesta of weaned piglets, while the addition of AX to the diet significantly increased an abundance of *Lactobacillus* in the ileal digesta and an abundance of *Bifidobacterium* in the colonic digesta of weaned piglets. In addition, the XOS group did not affect abundances of *Lactobacillus* and *Bifidobacterium*. The results mentioned above indicate that XOS could be almost fermented by gut microbiota in the upper intestine, leading to no changes in abundances of colonic *Lactobacillus* and *Bifidobacterium*. However, the AX could flow into the hindgut to be further fermented, resulting in an increased population of *Bifidobacterium.* Pang et al. [30] have reported that abundances of *Lactobacillus* and *Bifidobacterium* were markedly increased when weaning piglets were fed a diet containing 1.5% XOS. However, the high level of XOS did not markedly vary *Bifidobacterium* abundance in weaning piglets compared to rodents [22], probably due to the differences of microbial stability and physiological backgrounds between animal species. Evidence has shown that populations of *Lactobacillus* and *Bifidobacterium* are associated with SCFA production, antioxidant activity, and host inflammatory status [30,31,32,33,34,35]. Thus, the lower diarrhea incidence and improved immune function or antioxidant capacity when weaned piglets were fed the 1% XOS or 1% AX should be associated with the increased abundances of *Lactobacillus* and *Bifidobacterium* with increasing production of organic acids in the intestine of pigs.

A previous study showed dietary supplementation of XOS with different inclusion doses from 0% to 3% linearly increased the fecal acetate, propionate, butyrate, and total SCFA concentration in weaning piglets [30]. Chen et al. [2] also found that the dietary supplementation of 500 mg/kg XOS elevated the acetate, propionate, butyrate, and total SCFA concentrations in the cecum of piglets. The results mentioned above were consistent with our findings that the diet supplemented with 1% XOS significantly increased the lactic acid concentration in the ileum compared with the AX, but no improvement of colonic SCFA concentrations was found when weaned pigs were fed the XOS and AX. The different results for effects of AX or XOS on SCFA concentration in the hindgut of pigs between our finding and other publications can be associated with SCFA absorption by the intestinal epithelial cells.

In the present study, the addition of XOS or AX to the diet significantly increased the contents of lactic acid, acetic acid, and total organic acids in the ileum of weaned piglets. Similarly, in vitro fermentation of AX or XOS significantly increased the contents of acetic acid, propionic acid, butyric acid, and total organic acids, and the proportions of propionic acid and butyric acid to total organic acid were greater in the AX and XOS groups than those in the control group. In addition, in vitro fermentation of XOS showed a faster reduction of pH than the AX in the present study, which indicated XOS with a smaller molecular mass and a lower polymerization can be fermented by gut microbiota quickly compared with the AX. In addition, there were no differences in the concentrations of SCFA between the in vitro assays of the XOS and AX groups, which was associated with the fermentibility of XOS and AX whereby both XOS and AX can be almost fully fermented by gut microbiota in vitro [36,37].

## 5. Conclusions

In summary, both XOS and AX could reduce diarrhea incidence and improve antioxidant activity, sIgA and IL-10 concentrations, populations of *Lactobacillus* and *Bifidobacterium*, and SCFA production in the intestine of weaned pigs, but they have no effects on pig growth performance. In addition, XOS can be fermented in the upper gut of weaned pigs and produce more organic acids compared with AX, indicating XOS is fermented faster by gut microbiota than AX, which is associated with the smaller molecular mass of XOS.

## Data Availability

The data that support the findings of this study are available from the corresponding author upon reasonable request.

## Supplementary Materials

The following supporting information can be downloaded at: https://www.mdpi.com/article/10.3390/ani13060964/s1, Table S1: Composition and nutrient levels of the control diet (%, as-fed basis); Table S2: The criteria for fecal score; Figure S1: Effects of xylo-oligosaccharides and arabinoxylans on intestinal morphology in the ileum and colon of weaned pigs.
