# Supplementary material for: Effect of Arabinoxylan and Xylo-Oligosaccharide on Growth Performance and Intestinal Barrier Function in Weaned Piglets
Source: Animals (Basel). 2023 Mar 7;13(6):964. doi: 10.3390/ani13060964 (PMC10044045; doi:10.3390/ani13060964)
Supplement: Supplementary file 1 [file animals-13-00964-s001.zip › animals-2236402-supplementary.pdf]

Table S1. Composition and nutrient levels of the control diet (%, as-fed basis)

Items	Control diet	XOS diet	AX diet
Corn	56.45	55.05	55.05
Soybean meal	12.50	12.70	12.70
Whey powder	10.00	10.00	10.00
Fish meal	4.00	4.00	4.00
Soy protein concentrate	5.00	5.00	5.00
Extruded full-fat soybean	5.00	5.00	5.00
Xylo-oligosaccharide (XOS)	-	1.00	-
Arabinoxylan (AX)	-	-	1.00
Sucrose	2.00	2.00	2.00
Soybean oil	1.30	1.50	1.50
Dicalcium phosphate	1.20	1.20	1.20
Limestone	0.75	0.75	0.75
NaCl	0.20	0.20	0.20
L-Lysine-HCl	0.45	0.45	0.45
DL-Methionine	0.20	0.20	0.20
L-Threonine	0.15	0.15	0.15
L-Tryptophan	0.10	0.10	0.10
L-Valine	0.20	0.20	0.20
Vitamins and trace minerals[1]	0.50	0.50	0.50
Nutrient levels, %			
Calculated values			
Digestible energy, MJ/kg	14.80	14.80	14.80
Crude protein	19.00	19.00	19.00
Analyzed values, %			
Crude protein	18.85	18.93	18.77
Total dietary fiber	16.27	16.73	17.04
Soluble dietary fiber	1.97	2.34	2.14
Insoluble dietary fiber	14.30	14.39	14.90
Neutral detergent fiber	13.87	13.81	13.03
Acid detergent fiber	4.48	4.52	4.24

Notes: [1]Premix provided the following per kilogram of feed: vitamin A, 10,000 IU; vitamin D_3, 2,500 IU; vitamin E, 30 IU; vitamin K_3, 3 mg; vitamin B_1, 2.5 mg; vitamin B_2, 4.0 mg; vitamin B_6, 3.0 mg; vitamin B_{12}, 12 μg; nicotinic acid, 40 mg; thiamine, 3 mg; Riboflavin, 6 mg; D-pantothenic acid, 15 mg; folic acid, 1.2 mg; biotin, 50 μg; Fe, 90.0 mg; Zn, 75.0 mg; Mn, 40.0 mg; I, 0.35 mg; Se, 0.3 mg.

Table S2. The criteria for fecal score

Fecal score	Fecal morphology
Grade 1	hard, dry, and friable feces
Grade 2	normal feces
Grade 3	pasty feces, mild diarrhea
Grade 4	unfoemed feces, moderate diarrhea
Grade 5	watery feces, severe diarrhea

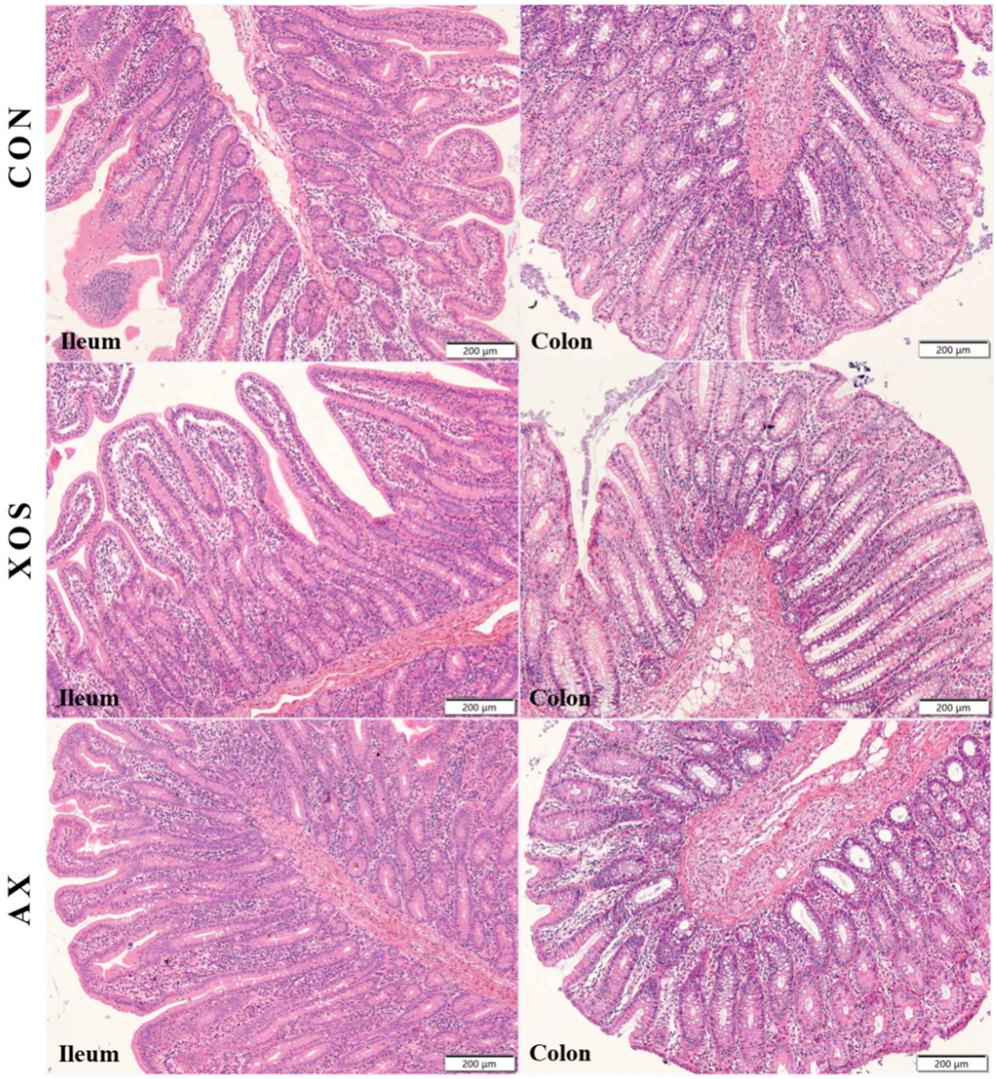

Figure S1. Effects of xylo-oligosaccharides and arabinoxylans on intestinal morphology in the ileum and colon of weaned pigs. n=6. Note: CON, a control diet; XOS, a diet containing 1% xylo-oligosaccharide; AX, a diet containing 1% arabinoxylans
